# Impact of Transmitter Positioning and Orientation Uncertainty on RSS-Based Visible Light Positioning Accuracy †

**DOI:** 10.3390/s21093044

**Published:** 2021-04-27

**Authors:** Neha Chaudhary, Luis Nero Alves, Zabih Ghassemlooy

**Affiliations:** 1 Instituto de Telecomunicações and Departamento de Electrónica, Telecomunicações e Informática, Universidade de Aveiro, 3810-193 Aveiro, Portugal; nero@ua.pt; 2 Optical Communications Research Group, Faculty of Engineering and Environment, Northumbria University, Newcastle upon Tyne NE1 8ST, UK; z.ghassemlooy@northumbria.ac.uk

**Keywords:** localization, VLC, visible light positioning, received signal strength, localization algorithm, Tx’s uncertainty

## Abstract

This paper present simulation-based results on the impact of transmitter (Tx) position and orientation uncertainty on the accuracy of the visible light positioning (VLP) system based on the received signal strength (RSS). There are several constraining factors for RSS-based algorithms, particularly due to multipath channel characteristics and set-up uncertainties. The impact of Tx uncertainties on positioning error performance is studied, assuming a statistical modelling of the uncertainties. Simulation results show that the Tx uncertainties have a severe impact on the positioning error, which can be leveraged through the usage of more transmitters. Concerning a smaller Tx’s position uncertainty of 5 cm, the average positioning errors are 23.3, 15.1, and 13.2 cm with the standard deviation values of 6.4, 4.1, and 2.7 cm for 4-, 9-, and 16-Tx cases, respectively. While for a smaller Tx’ orientation uncertainty of 5°, the average positioning errors are 31.9, 20.6, and 17 cm with standard deviation values of 9.2, 6.3, and 3.9 cm for 4-, 9-, and 16-Tx cases, respectively.

## 1. Introduction

The demand for highly precise indoor positioning (IP) systems is growing rapidly due to its potential in the increasingly popular techniques of Internet of Thing (IoT), smart mobile devices, and artificial intelligence. Consequently, IP becomes a promising research domain that is getting wide attention due to its benefits in several working scenarios, such as, industries, health sectors, indoor public locations, and autonomous navigation [1]. The traditional positioning methods, which depend on satellites such as the global positioning system (GPS), is common nowadays for outdoor positioning; however, it is not well-suited for indoor environments. This is because the GPS signals from the satellites suffer from high penetration loss and multipath fading due to building walls. Some other systems also have been proposed for IP, for example, Bluetooth, ultrasound, ultra-wideband (UWB), wireless-Fidelity, radio frequency identification (RFID), and radio-frequency (RF)-based techniques [2,3,4]. For instance, the UWB technology transmits short RF pulses with a low duty cycle, which provides precise localization and tracking of mobile devices in indoor environments [4]. Despite the advantage of precise localization, the UWB technology is still not perfect for IP systems, and it has not been embraced widely because of its cost, complexity, and need for synchronization between transmitters (Txs) and the targets [5]. Moreover, these RF-based systems may not be suitable in RF restricted areas, such as hospitals due to the RF-induced interference [2].

Another developed technology that makes use of the pre-installed lighting infrastructure is visible light positioning (VLP). VLP is based on visible light communications (VLC), which is license-free, and free from RF induced electromagnetic interference-free, thus making it ideal in many applications including hospitals. In addition, VLC uses the pre-existing light-emitting diodes (LEDs) infrastructure as a Tx which has the ability to provide illuminance and communication simultaneously [6]. VLP is an emerging technology promising high accuracy, high security, low deployment cost, shorter time response, and low relative complexity when compared with RF-based positioning [7].

Existing VLP approaches estimate the position of a receiver (Rx) based on several characteristics of light including received signal strength (RSS) [8], angle of arrival (AOA)/angle difference of arrival (ADOA) [9], image [10], and time of arrival (TOA)/time difference of arrival (TDOA) [11]. It has been established that, VLP based on AOA/ADOA, image, and TOA/TDOA require auxiliary devices to capture angle/image/time. On the contrary, the RSS approach can be achieved by utilizing a single photodiode (PD) without the need of any additional auxiliary devices, which makes the RSS-based approach the most well-known method for VLP [12].

Recent research works have addressed the impact of (*i*) LED power uncertainty [13]; (*ii*) reflections from walls and objects within the transmission paths [14]; and (*iii*) noise on the positioning performance [15]. The multipath channel has a direct influence on the model for the estimation of the received signal power, which has been addressed previously using machine learning algorithms [16]. The Tx and the Rx design specifications, such as the Tx beam width and its tilting angle have been investigated before. For instance, in [13], the impact of LED output power uncertainty on the accuracy of the RSS-based VLP system was explored, with the maximum error of 17 and 40 cm for a tolerance (possible variations) value of 5% and 20%, respectively. The performance analysis of various VLP systems relies on line-of-sight (LOS) transmission path, which can underestimate the achievable error bounds, due to the fact that a real scenario will definitely include non-line of sight (NLOS) paths [14]. Therefore, NLOS transmission paths should not be neglected. In [15], an RSS-based VLP system using received optical power from the emitting LEDs was investigated considering signals from both LOS and NLOS paths. The results revealed that, the positioning accuracy of <10 cm on average can be achieved at a signal-to-noise ratio (SNR) of >12 dB.

The location of the Rx can be estimated by using different estimation methods, such as linear least square (LLS) and non-linear least square (NLLS) [14]. In [17], a polynomial regression-based method was used to investigate the accuracy of an RSS-based VLP system along with LLS and NLLS estimation algorithms. The results revealed that, the positioning error ε*_p_* was <0.6 m by using the regression approach, which is much lower than other traditional methods. In [18], the impact of the Tx’s orientation (i.e., the tilting angle) on the positioning accuracy of the RSS-based VLP system was studied. Another estimation method, i.e., normalized least square estimation was utilized to estimate the positioning accuracy. In [19], an artificial neural network (ANN) based 4-LED VLP system was proposed to reduce ε*_p_* for the LOS path, which is affected by the random and unknown static Tx tilt angle with a maximum variation of 2°. It was revealed that ANN achieved localization errors below 1 cm. In general, the positions and orientations of Txs may not be symmetrical, which (*i*) depends on the indoor environments such as museums, galleries, train stations, shopping centers, etc., where lights are pointing in different directions; and (*ii*) can change when replacing lights, carrying out maintenance, etc. Therefore, the random variations in the Tx’s orientation will lead to the random errors in VLP systems, which requires further studies. The impact of Tx’s position and its orientation uncertainties on the positioning estimation have not yet been systematically explored, which is the objective of this paper.

In this paper, we investigate the impact of Tx’s position and its orientation uncertainties on ε*_p_* of the RSS-based VLP system under multipath reflections. The uniform distribution of light inside the illuminated place is a necessity in indoor environments. As a result, lighting uniformity becomes a vital aspect for a well-lit environment. Moreover, both lighting uniformity and the positioning performance are related to the Tx’s positions and the Lambertian half-power angles (HPA). Therefore, in this work we investigate (*i*) how the uniformity of light in the room changes for different HPA and the Tx positions; and (*ii*) the impact of Tx position and its orientation uncertainties on the positioning accuracy considering the optimized Tx positions from a lighting uniformity perspective. This works focused only on the LED uncertainties caused during installation and is an extension of our previous work [20], where the problem of Tx’s orientation uncertainty was not considered.

The rest of the paper is organized as follows, Section 2 introduces the set-up of the system and position estimation approaches in detail. The impact of different set-up uncertainties and uniformity are presented in Section 3. In Section 4, a discussion of the simulation results attained is made, followed by the final concluding remarks in Section 5.

## 2. VLP System Modelling

### 2.1. Channel Model

**Figure 1:**
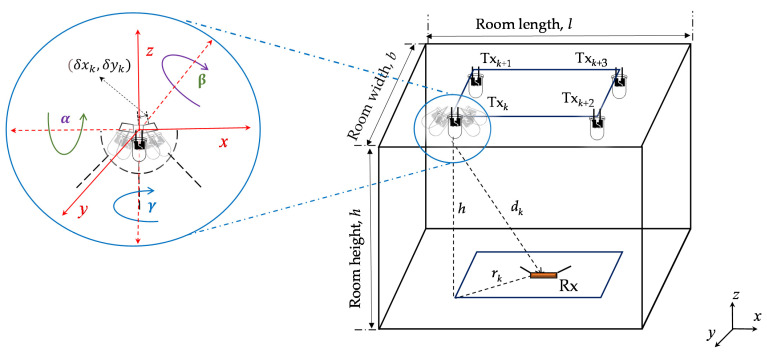
Overview of the simulation set-up.

Our proposed VLP system is composed of a PD as an Rx, which is placed on the ground and a number of LEDs as the Txs (i.e., 4-, 9-, and 16-LED) that are installed on the ceiling of the room as depicted in Figure 1. The field of view (FOV) and the detection area *A_r_* of the PD are 70° and 10^−4^ m^2^, respectively. All Txs are located at the same height *h* from the ground level and the coordinates of *k*th Tx (*k* = 1, …, *K*) is ($x_{k},y_{k},z_{k}$), where *K* is the total number of Txs. The Rx coordinate is denoted by ($x_{r},y_{r},z_{r}$). The position and orientation of the Txs is best illustrated by the ${\mathrm{Tx}}_{k}$, with the coordinate of (δx,δy) and the angles of α, β, and γ. Both δx and δy, and α, β, and γ, are assumed to be Gaussian variables with *N*(0, $\;\sigma^{2}$) and *N*(0, ζ^2^) probability distribution functions, respectively. The distance between the Txs relies on the lighting uniformity considerations that are described in a later section. An empty room is considered as a reference to study the impact of Tx’s position and its orientation uncertainty on the positioning accuracy. In this work, both LOS and NLOS transmission paths are assumed between the Txs and the Rx. However, for the NLOS path, we only consider the first reflection due to the fact that the second order reflections have much reduced intensities and therefore can be neglected [21]. Each Tx broadcast a unique 2-bit ID information, which is encoded and modulated using on-off keying (OOK), which allows separation at the Rx using a correlation method that can be received at the Rx in advance of location identification.

The total received optical power $P_{r}$ at the PD comprises the power from the LOS and NLOS paths, as given by:

(1) $$P_{r}=\sum P_{r\left(\mathrm{LOS}\right)}+\sum P_{r\left(\mathrm{NLOS}\right)},$$

where $P_{r\left(\mathrm{LOS}\right)}$ and $P_{r\left(\mathrm{NLOS}\right)}$ represent the received power for LOS and NLOS, respectively. Generally, the SNR will be high (i.e., >20 dB) in standard VLC, which could be considered noise-free in normal cases [22]. Thus, in this study, no actual noise (e.g., shot noise or thermal noise) is considered as a source of “measurement noise”, to unambiguously evaluate the effect of the Tx’s position and its orientation uncertainty.

### 2.2. RSS-Based Positioning

Using the RSS algorithm, $P_{r\left(\mathrm{LOS}\right)}$ for the LOS path can be expressed as [22,23]:

(2) $$\sum P_{r\left(\mathrm{LOS}\right)\;}=\overset{K}{\underset{k=1}{\sum }}P_{t}\;A_{r}ℛ\left(\frac{m+1}{2\pi }\right)\frac{\cos^{m}\left(\omega_{k}\right)\;cos\left(\varphi \right)}{{\left\|d_{k}\right\|}^{2}}T_{s}\left(\varphi \right)g\left(\varphi \right),$$

where

(3) $$m=-\frac{\ln\left(2\right)}{\ln\left(\cos\left(\Theta_{\frac{1}{2}}\right)\right)},$$

where $P_{t}$ is the transmit power, $d_{k}$ is the distance between *k*th Tx and the Rx, *K* is the total number of Txs, and $\Theta_{1/2}\;$ is the light source irradiance half-power angle (HPA) [22]. $\omega_{k}$ and *φ* are the irradiance angle from the *k*th Tx to the Rx and the receiving incident angle, respectively. *A_r_* and ℛ are the PD’s active area and responsivity, respectively. $T_{s}\left(\varphi \right)$ and g(φ) are the gains of the optical filter and the concentrator at the Rx, respectively. Note, $T_{s}\;\left(\varphi \right)$ and g(φ)  are set to unity.

**Figure 2:**
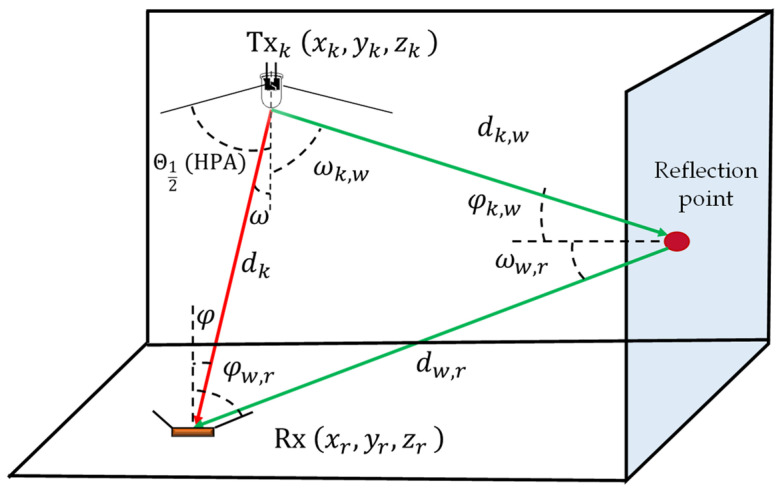
Overview of the visible light channel.

Considering the NLOS path, i.e., the first-order reflection, the total received power can be expressed as [22]:

(4) $$\sum P_{r\left(\mathrm{NLOS}\right)}=\overset{K}{\underset{k=1}{\sum }}\underset{\mathrm{wall}}{\sum }\rho P_{t}\;A_{r}ℛ\left(\frac{m+1}{2\pi }\right)A_{\mathrm{ref}}\frac{\cos^{m}\left(\omega_{k,w}\right)\;cos\left(\varphi_{k,w}\right)}{\pi {\left(\left\|d_{k,w}\right\|\left\|d_{w,r}\right\|\right)}^{2}}\;T_{s}\left(\varphi_{w,r}\right)\;g\left(\varphi_{w,r}\right)\cos\left(\omega_{w,r}\right)\cos\left(\varphi_{w,r}\right),\;\;\;$$

where $d_{k,w}$, $\varphi_{k,w\;}$, and $\omega_{k,w}$ are the distances, receiving incident angle, and the irradiance angle between the *k*th Tx and the reflective area, respectively. $d_{w,r}$, $\;\varphi_{w,r}$, and $\omega_{w,r}$ are the distances, receiving incident angle, and the irradiance angle between the reflective area and the Rx, respectively, see Figure 2. *ρ* is the reflection coefficient that relies on the reflective surface material, and $A_{\mathrm{ref}}$ is the reflection area. $P_{r\left(\mathrm{NLOS}\right)}\;$ for the signals from the NLOS paths is obtained based on Matlab [22]. For each Tx, we integrate (4) over all the walls accompanied by the assumption of a grid area with a resolution $A_{\mathrm{ref}}$ of 0.1 m.

### 2.3. Distance Estimation Using Polynomial Regression

**Figure 3:**
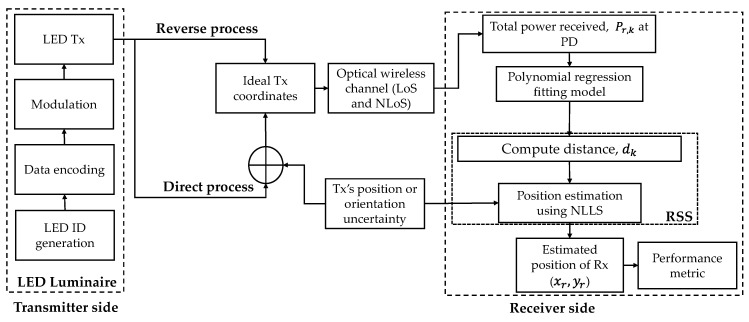
Block diagram of the proposed system.

The block diagram of the proposed VLP system is shown in Figure 3. In the case of LOS, the horizontal distance can be computed as ${\left\|r_{k}\right\|}^{2}={\left\|d_{k}\right\|}^{2}-h^{2}$ and therefore can be determined from (2) as given by:

(5) $$r_{k}=\sqrt{{\left(\frac{\left(m+1\right)\;A_{r}ℛP_{t}{\left(h\right)}^{m+1}}{2\pi P_{r\left(\mathrm{LOS}\right),k}}\right)}^{\frac{2}{m+3}}-h^{2},}$$

where *h* is the vertical distance between the Tx and the Rx. $P_{r\left(\mathrm{LOS}\right),k}$ is the LOS received power at the Rx from the *k*th Tx. In (5), the cosine terms are clearly stated in terms of the set-up geometry, as $h/\|d_{k}\|$. However, in case of NLOS links, high errors are introduced in the channel due to the presence of reflections [17,24], thus, the above method fails to determine the distance. Another method that can be used is to employ a polynomial fitted model for the power-distance relationship [25]. In this particular case, the relation between $d_{k}$ and $P_{r,\;k}$ for the *k*th Tx is represented as:

(6) $$d_{k}=\alpha_{0}+\alpha_{1}P_{r,k}+\alpha_{2}{\left(P_{r,k}\right)}^{2}+\ldots +\alpha_{s}{\left(P_{r,k}\right)}^{s}$$

where $P_{r,\;k}$ is the total received power at the Rx from the *k*th Tx, and $\alpha_{s}$ represent the coefficients of the fitted polynomial. Note, $d_{k}$ is computed using (6), which is later employed to determine $r_{k}$.

**Figure 4:**
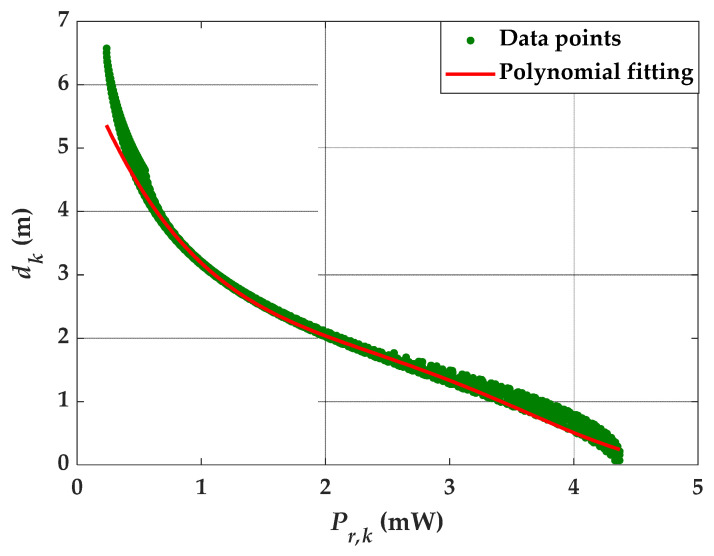
Estimation using the polynomial fitting method.

We estimated the best polynomial fitting using 3600 data points extracted from channel simulation, see Figure 4. Note that, the 3600 data points are considered as 3600 different locations each within the room. The best-fitted solution was exhibited by analyzing the value of *R*^2^ (coefficient of determination) for different orders, and a fourth order polynomial was selected with a value of *R*^2^ of 0.8942. Note, the polynomial fitting is not highly accurate. This is because of the data points considered within the entire room for both LOS and NLOS paths. At the center of the room, the impact of NLOS is negligible when compared to the regions near walls and corners. As such, fitting all the data will be dominated by the data near walls and corners. However, it shows the best fitted solution considering that the data points corresponding to lower received power have higher contributions to the error compared with the data points in the center of the room. The values of polynomial coefficients are listed in Table 1.

### 2.4. Estimation Using Nonlinear Least Squares

The relation between the Tx coordinates ($x_{k},y_{k}$) and the Rx coordinates ($x_{r},y_{r}$) are described using the following equations:

(7) $$\{\begin{array}{c} {\left(x_{r}-x_{1}\right)}^{2}+{\left(y_{r}-y_{1}\right)}^{2}=r_{1}{}^{2}\\ \begin{array}{c} {\left(x_{r}-x_{2}\right)}^{2}+{\left(y_{r}-y_{2}\right)}^{2}=r_{2}{}^{2}\\ \vdots \end{array}\\ {\left(x_{r}-x_{K}\right)}^{2}+{\left(y_{r}-y_{K}\right)}^{2}=r_{K}{}^{2} \end{array},$$

where *K* is the total number of Txs. NLLS estimation can be utilized to estimate the target location, in which the solution can be estimated by attaining $\tilde{X}=\left[\tilde{x},\tilde{y}\right]$ that minimizes a cost function given by [15]:

(8) $$\tilde{Q}=\underset{i}{\sum }{\left(\sqrt{{\left(x_{r}-x_{k}\right)}^{2}+{\left(y_{r}-y_{k}\right)}^{2}}-r_{k}\right)}^{2}.$$

An iterative procedure is utilized to estimate $\tilde{X}$ by employing the trust-region reflective algorithm [26]. In this algorithm, first, an estimate is introduced as ${\tilde{X}}_{0}$, followed by computing the corresponding cost function ${\tilde{Q}}_{0}$. Next, several points in the neighborhood of ${\tilde{X}}_{0}$ are replaced in (8), and the one that minimizes the cost ${\tilde{Q}}_{1}$ is selected as ${\tilde{X}}_{1}$. The Rx coordinates $\tilde{X}$ will eventually be obtained following several iterative steps to ensure convergence of $\tilde{Q}$. In the proposed system, the initial value for ${\tilde{X}}_{0}\;$ is estimated using a linear least square approach.

### 2.5. Performance Metrics

ε*_p_* is assumed to be a random variable (as it may rely on the uncertainties, i.e., the Tx’s position or its orientation uncertainties, estimation process, or noise); thus, it is reasonable to use the standard statistical analyses to access error performance. Here we use the probability distribution function (PDF) and the cumulative distribution function (CDF), as a mean to calculate the 95% quantile on ε*_p_*. Hence, the PDF and CDF are composed of the spatial distribution of ε*_p_* within the entire room. The PDF of ε*_p_* is defined as:

(9) $$f_{\varepsilon_{p}}\left(p\right)=\underset{\begin{array}{c} N\to \infty \\ \varepsilon \to 0 \end{array}}{\lim}\frac{\#\varepsilon_{p}\left(\left|\varepsilon_{p}-p\right|\leq \varepsilon \right)}{N},$$

where *ε* defines an error interval centered around *p*, and *N* is the number of samples. The cardinal operator *#* signifies the counting of occurrences were *|*$\varepsilon_{p}$*-p| < ε*. The limiting process is naturally implied by the discrete nature of the simulation. The CDF can be expressed as:

(10) $$F_{\mathrm{CDF}}\left(p\right)=\overset{p}{\underset{0}{\int }}f_{\varepsilon_{p}}\left(p\right)dp.$$

**Figure 5:**
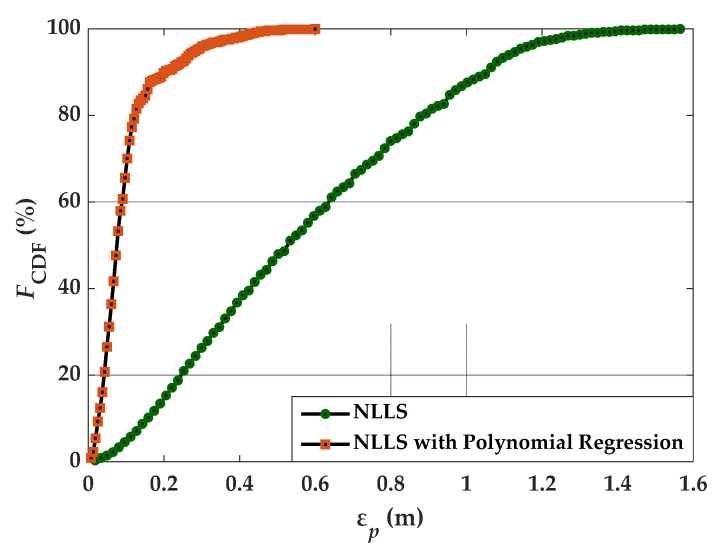
CDF of the ε*_p_* computed by NLLS and NLLS with polynomial regression estimation.

Figure 5 depicts the CDF against ε*_p_* for NLLS estimation with and without polynomial regression. The maximum ε*_p_* values estimated by NLLS and NLLS with polynomial regression are 0.6 and 1.57 m, respectively. It is observed that, there is an evident improvement in the positioning accuracy by using NLLS estimation with polynomial regression for power-distance modeling. Therefore, the polynomial regression can improve the accuracy of position estimation without the inclusion of high complexity algorithms. However, there are some limitations of the polynomial regression; for instance, the coefficients of the polynomial model must be provided along with the Tx’s positions in practical scenarios. Moreover, the polynomial model can deal with the empty and non-empty rooms with the fixed furniture and objects, but with no user’s mobility.

## 3. Set-Up Uncertainties

The major challenge in studying the Tx’s position or orientation uncertainties occurs in simulation, i.e., we must use random Tx positions in order to investigate the uncertainties. This raises the necessity to re-simulate the channel for each iteration (new random TX position). Give that the channel estimation is by itself complex and time-consuming, this problem becomes complex. We have developed two approaches for both Tx’s position and its orientation uncertainties to fully analyze the problem, which is termed direct and reverse processes. The reason for these designations is straightforward. The direct process entails the estimation of the position given the effect of the uncertainties on the channel. It is in this sense, complex and time-consuming. In the reverse process, the channel is assumed fixed and the effects of the Tx uncertainties are coupled directly into the estimation equations, this avoids the channel simulation at each set of newly generated Tx positions.

### 3.1. Uncertainties of the Tx’s Position

For Tx’s position uncertainties, in the direct process, we assume that the channel is re-stimulated for each newly generated set of Tx positions. The Tx positions are composed by the ideal position (fixed) and a random perturbation in two-dimensions. While in the reverse process, we assume that the channel is fixed for a set of ideal Tx positions, and the uncertainties are directly coupled into the estimation Equation (7). To illustrate the concept, let us consider one of the terms in (7), in the direct process, the effect of the uncertainties is on the right side of the equation, as follows:

(11) $${\left(x_{r}-x_{k}\right)}^{2}+{\left(y_{r}-y_{k}\right)}^{2}=f_{R}\left(x_{k}+\delta x,y_{k}+\delta y\right),$$

where ($x_{r},y_{r}$) and ($x_{k},y_{k}$) are the coordinates of the Rx and the Tx (ideal positions), respectively. (δx,δy) express the uncertainty at the Tx coordinates. Both δx and δy are assumed to be Gaussian variables with *N*(0, $\;\sigma^{2}$). The channel needs to be re-simulated at each iteration in order to obtain *f_R_*.

In the reverse process, it is not required to re-simulate the channel at each iteration as the uncertainties are part of the Tx position, as follows:

(12) $${\left(x_{r}-x_{k}-\delta x\right)}^{2}+{\left(y_{r}-y_{k}-\delta y\right)}^{2}=f_{R}\left(x_{k},y_{k}\right).$$

Subtracting (11) and (12) and extracting the mathematical expectation of both sides gives:

(13) $$U_{r}\left(\sigma_{r}\right)=E\left[\delta x^{2}+\delta y^{2}-2\delta x\left(x_{r}-x_{k}\right)-2\delta y\left(y_{r}-y_{k}\right)\right],$$

where $\sigma_{r}$ is the Tx’s uncertainty of the reverse process. The reverse process has an expectation as stated in (13), which is given by (after solving (13)):

(14) $$U_{r}\left(\sigma_{r}\right)=2\sigma_{r}{}^{2}.$$

Performing the same analysis for the direct process we have:

(15) $$U_{d}\left(\sigma_{d}\right)=E\left[f_{R}\left(x_{k},y_{k}\right)-f_{R}\left(x_{k}+\delta x,y_{r}+\delta y\right)\right].$$

**Figure 6:**
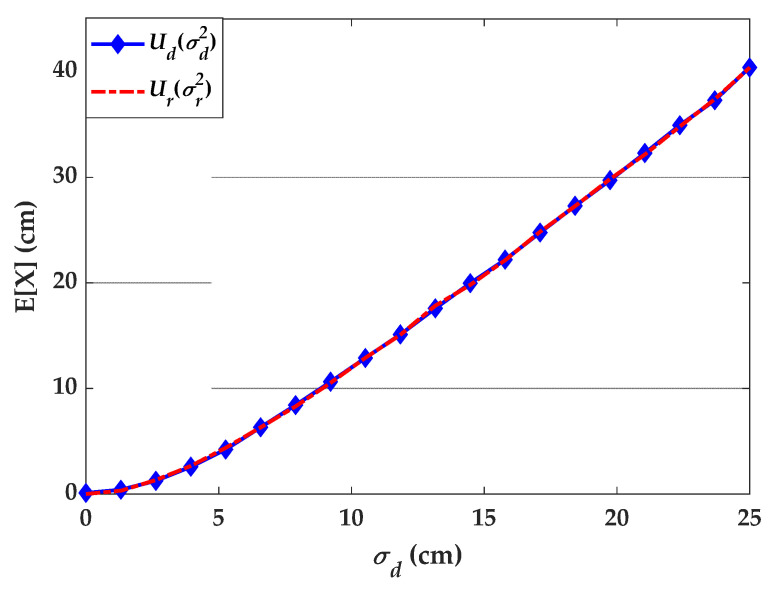
Study on Tx’s position uncertainty based on the direct and reverse processes.

Here, the value of $U_{d}$ is estimated using numerical simulation, where $\sigma_{d}$ is the Tx’s uncertainty of the direct process. Assuming that, the reverse and direct processes are equivalent, the expectation values $U_{d}$ and $U_{r}$ must be equal. In this case, it is possible to infer the relation between $\sigma_{d}$ and $\sigma_{r}$. This relation can be estimated assuming that the Rx is placed at the center of the room and a single Tx is placed in a uniform grid of positions for the whole room. Using mathematical simulation, we have computed the values of $U_{d}\left(\sigma_{d}\right)$ for different values of $\sigma_{d}$, see Figure 6. Note, the two processes generate uncertainties, i.e., $U_{d}$ and $\;U_{r}$ and these processes would be equivalent if these uncertainties are the same for certain values of $\sigma_{d}$ and $\sigma_{r}$. This indicates that, the uncertainties from the reverse process can be generated with the knowledge of the direct process as given by:

(16) $$\sigma_{r}^{2}=\frac{U_{d}\left(\sigma_{d}\right)}{2}.$$

Simulating the uncertainty for the reverse process using the correction in (16) provides the same values for the expectation as depicted in Figure 6 (curve labelled $U_{r}$).

### 3.2. Uncertainties of the Tx’s Orientation

The Tx’s orientation uncertainties can be treated in a similar fashion as before. There are, however, some aspects that need addressing. In this paper, the Tx’ orientation uncertainties are modeled via three random axis-rotations of the Tx’s heading vector. Let us consider the *k*th Tx’s heading vector, $\hat{n_{k}}$, which is given by:

(17) $$\hat{n_{k}}=R_{x}\left(\alpha \right)\cdot R_{y}\left(\beta \right)\cdot R_{z}\left(\gamma \right)\cdot \hat{n_{k}^{0}}=R\left(\alpha ,\;\beta ,\gamma \right)\cdot \hat{n_{k}^{0}}$$

where $R_{x}\left(\alpha \right)$, $\;R_{y}\left(\beta \right)$ and $R_{z}\left(\gamma \right)$ represent the three rotation matrix relative to the *x*-axis, *y*-axis, and *z*-axis, respectively, α, β, and γ are three random angles with probability distribution function *N*(0, ζ^2^), finally, $\hat{n_{k}^{0}}$ represents the unperturbed heading vector of the *k*th Tx. R(α, β,γ) is a composed rotation matrix. The effect of (17) on the Tx’s heading is due to the cosine terms due to the Txs in (2) and (4), showing that a rotation will impact the received signal. The next step resorts to the evaluation of the expectations due to the direct and reverse process. In the direct process, the channel is re-simulated for each random iteration, generating an expectation value, for the *k*th Tx, which is given by:

(18) $$U_{d}\left(\zeta_{d}\right)=E\left[f_{R}\left(x_{k},y_{k}\right)-f_{R}\left(x_{k},y_{r},R\left(\alpha ,\beta ,\gamma \right)\right)\right].$$

**Figure 7:**
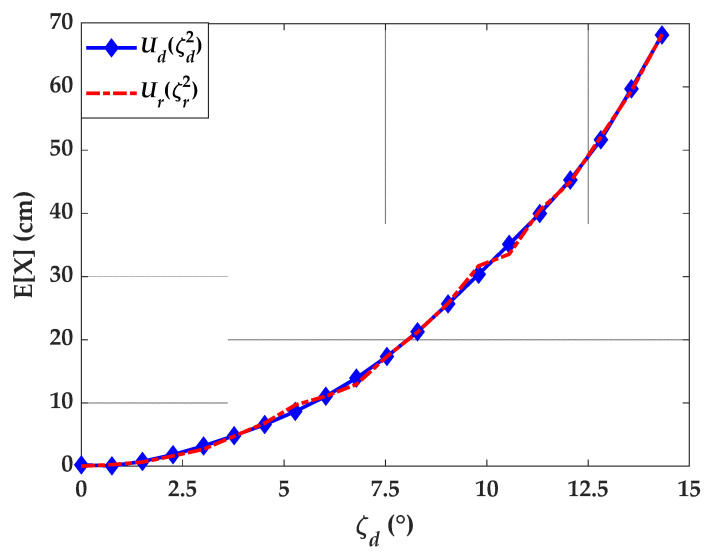
Study on Tx’s orientation uncertainty based on the direct and reverse processes.

$U_{d}\;$ cannot be cast in a closed-form solution. Instead, we resorted to simulation using the approach described in the previous section, results are depicted in Figure 7.

The expectation of the reverse process reveals some difficulties. The quadratic forms in the left side of (7) are invariant to rotations, which would imply that orientation uncertainties do not affect error performance. Since this is not the case, we approach the problem in a similar manner as in the case of Tx’s position uncertainties, that is, assuming that the effect of random headings can be reproduced by affecting the Tx’s positions with a random perturbation. Following this approach, the expectation of the reverse process can be cast as:

(19) $$U_{r}\left(\zeta_{r}\right)=E\left[\delta x^{2}+\delta y^{2}-2\left(x_{r}-x_{k}\right)\delta x-2\left(y_{r}-y_{k}\right)\delta y\right]=2\zeta_{r}{}^{2}$$

where δx and δy are the equivalent position perturbations, which are functions of the random orientation angles α, β, and γ. Equating $U_{d}$ and $\;U_{r}$, allows to retrieve the correction values for the angle uncertainty, $\zeta_{r}$ as a function of $\zeta_{d}$. To show that the processes are indeed equivalent, we performed the simulation for $U_{r}$, the results are depicted in Figure 7. As it can be seen the two expectations match well and reveal that the effect of orientation uncertainty is worse than the position uncertainty.

### 3.3. Lighting Uniformity

In the indoor environments, it is essential to uniformly distribute $P_{r}$ inside the illuminated zone [27]. This constraint is generally assumed in well-lit spaces, where the uniformity of light is linked to the best perception of the objects. The uniform distribution of light in the room, $U_{l}$, is represented as the ratio of the minimum to maximum power intensity at the receiving plane, which is given by:

(20) $$U_{l}=\frac{\min\left(P_{r}\right)}{\max(P_{r})},$$

**Figure 8:**
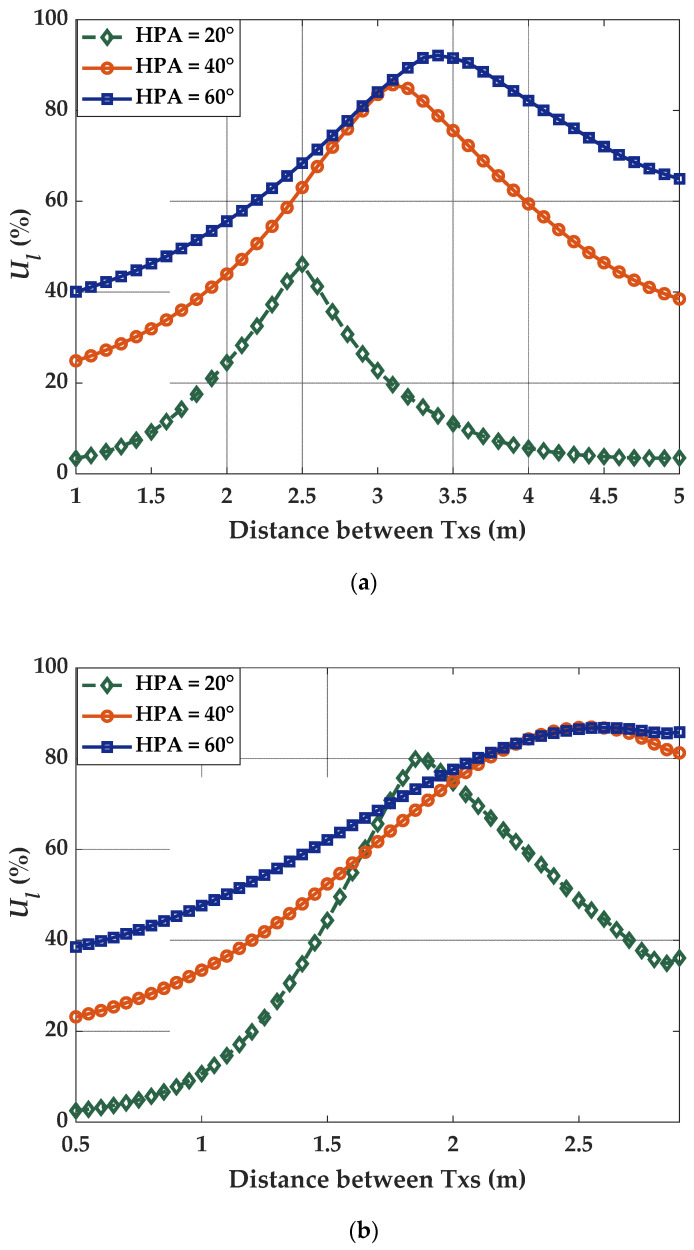
Comparison of uniformity for different HPA and distance between the Txs in case of (**a**) 4-Tx, and (**b**) 9-Tx.

Lighting uniformity relies on the three factors related to the Txs, i.e., the distance between the Txs, HPA, and the number of Txs. Figure 8 illustrates the lighting uniformity as a function of the number of Txs (i.e., 4 and 9) and for different values of HPA.

It is noticed from the figure that, a higher number of Txs provide improved uniformity. The maximum uniformity is achieved at distances of 3.4 and 2.6 m for HPA of 60° for 4- and 9-Tx, respectively. It is interesting to notice that, the same factors influence both lighting uniformity and positioning accuracy. It is usually believed that, positioning accuracy can be enhanced with higher uniformity. However, our study reveals that, this is not a general rule, moreover, HPA seems to have a more prominent impact on the positioning accuracy than the uniformity.

## 4. Simulation Results

In this section, the performance of the proposed VLP system is evaluated by simulation results. We consider the scenario where the LED-based Txs are located on the ceiling of a room of dimension 6 × 6 × 3 m^3^, which are assumed to be the same and modelled as pointwise Lambertian sources with order *m* depending ζ on the value of HPA, see (2). In practical environments also, the square LED placement layout is common. We have considered three different cases of 4-, 9-, and 16-LEDs, which are arranged in a square grid on the ceiling plane. The Rx is located on the ground plane. For all simulation purposes, the resolution of the grid is fixed at 1 cm, which implies that the PD can be placed at 3600 different locations. All the key parameters for the simulation are detailed in Table 2.

### 4.1. Positioning Error Dependence on Lighting Uniformity

**Figure 9:**
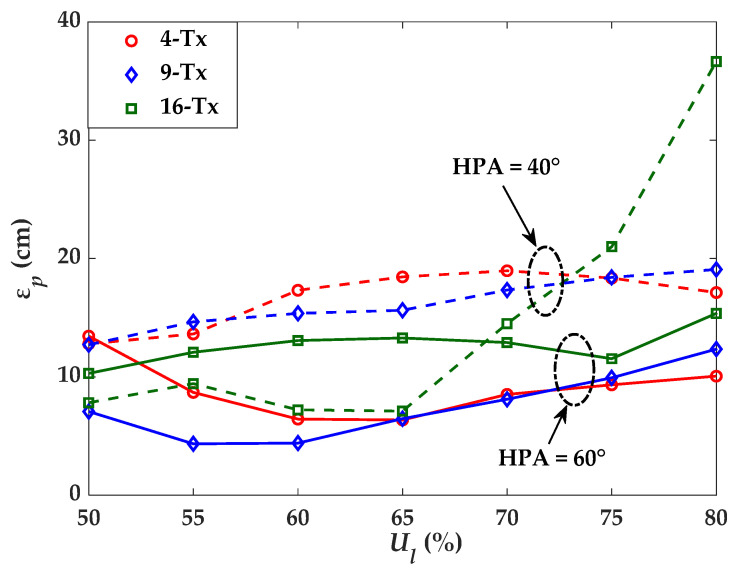
Comparison of ε*_p_* for different Txs and uniformity.

This section analyses the influence of lighting uniformity and HPA on ε*_p_*. Here, we vary the uniformity between 0.5 and 0.8 in steps of 0.05 and for HPA of 40° and 60°. For this, we first select the values of distance between the Txs according to the results of Figure 8. Following this, we estimate ε*_p_* for each set of conditions using NLLS and the polynomial regression power-distance model. ε*_p_* for different values of uniformity for 4-, 9-, and 16-Tx are illustrated in Figure 9. It is observed that the minimum ε*_p_* is attained for the case of 4-Tx with HPA and uniformity of 60° and 0.65, respectively, whereas, in the case of 9-Tx, the minimum ε*_p_* is achieved with HPA of 60° and uniformity equal to 0.55. For the 16-Tx case, the minimum ε*_p_* is accomplished with an HPA of 40° and with the uniformity of 0.65. It is clear from Figure 9 that, (*i*) low to a moderate value of lighting uniformity can support low ε*_p_*; and (*ii*) an optimal value for the number of Txs and the associated HPA, which do not match the optimal value of lighting uniformity conditions. Under the simulated conditions, the optimal values for the HPA, uniformity, and distance between the Tx based on the lowest ε*_p_* are shown in Table 3.

### 4.2. Impact of Tx’s Position and Orientation Uncertainty on Error Performance

In this section, we investigate the impact of Tx’s position uncertainties σ and the Tx’s orientation uncertainties ζ on the error performance. We follow the same approach that has been described in detail in Section 3.1. The simulation follows the reverse method with 1000 random iterations for each σ value using the adjusted variance values given by (16), where a random displacement vector (δx,δy) is added in the ideal Tx positions as explained in (11). The quantile function $Q_{F}\left(p\right)$ is employed as a performance metric considering both, its average and standard deviation, over the 1000 random iterations to obtain the confidence interval of ε*_p_*, which is given by:

(21) $$Q_{F}\left(p\right)=F_{\mathrm{CDF}}{}^{-1}\left(p\right),$$

where p is the percentage of the confidence interval.

**Figure 10:**
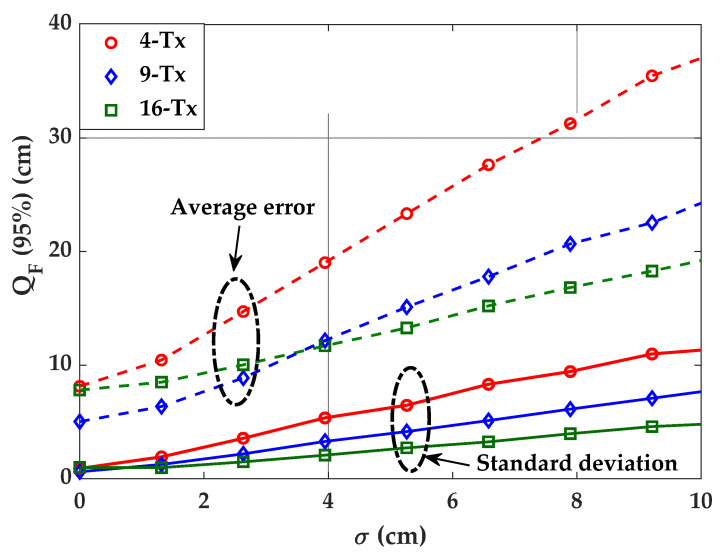
Comparison of positioning error for different Txs and σ.

Figure 10 illustrates the average and standard deviation of the 95% quantile of ε*_p_* as a function of σ for 4-, 9-, and 16-Tx for the Tx’s positioning uncertainty. It is observed that the effect of Tx’s position uncertainty, σ, traduces in an increasing error dependence, which is more prominent for set-ups with a lower number of Txs. The standard deviation of the error quantile, i.e., the increasing error also confirms an increasing trend with σ, is more evident for the 4-Tx case. It is noticed that, for σ of 5 cm the average positioning errors are 23.3, 15.1, and 13.2 cm, with the standard deviation values of 6.4, 4.1, and 2.7 cm for 4-, 9-, and 16-Tx cases, respectively. Figure 10 suggests that the error dependence on the Tx’s position uncertainty can be lowered by increasing the number of Txs.

**Figure 11:**
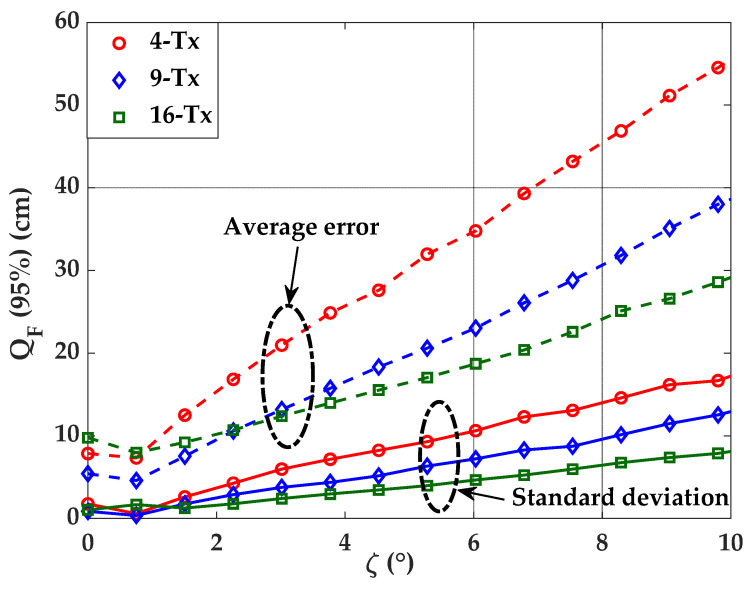
Comparison of positioning error for different Txs and ζ.

Figure 11 depicts the average and standard deviation of the 95% quantile of ε*_p_* or as a function of ζ for 4-, 9-, and 16-Tx for the Tx’s orientation uncertainty. It is clear from the figure that, alike the case of Tx’s position uncertainty, the effect ζ has more significant error dependence for set-ups with a lower number of Txs. It is observed that for ζ of 5°, the average positioning errors are 31.9, 20.6, and 17 cm, with the standard deviation values of 9.2, 6.3, and 3.9 cm for 4-, 9-, and 16-Tx cases, respectively. The increasing error also proves an increasing trend with ζ, therefore, the placement of Tx with the accurate Tx’s position and its orientation should be taken into consideration, as the error may rise even with low values of set-up uncertainties.

## 5. Conclusions

In this paper, we demonstrated the influence of Tx’s position and orientation uncertainty on the performance of VLP systems based on RSS. From the results, we can conclude that light uniformity and Tx’s HPA are crucial design parameters for developing an efficient VLP system. The selection of the Tx’s HPA as well as the optimum distance between Txs have to be carefully implemented. Moreover, we showed that the best uniformity and optimum error performance were not met for the same conditions, inferring necessary design trade-offs. Furthermore, the effect of error dependence on Tx’s position and orientation uncertainty reduced with increasing the number of Txs. In case of square grid Tx placement for a VLP system, the number of Txs can be further explored as an added variable to optimize both light uniformity and error performance, as it reduces the HPA for smaller distances between Txs.

## Figures and Tables

**Table 1:** The value of polynomial coefficients for the best polynomial fitting.

Polynomial Coefficient	$\alpha_{0}$	$\alpha_{1}$	$\alpha_{2}$	$\alpha_{3}$	$\alpha_{4}$
Value	$8.85\times 10^{6}$	$-9.93\times 10^{5}$	$3.96\times 10^{4}$	$-7.34\times 10^{2}$	7.4

**Table 2:** The key parameters for the indoor VLP system.

Parameter	Value
Room size	6 × 6 × 3 m^3^
Number of LED Txs	4/9/16
Transmit power of each Tx	1 W
Rx’s field of view	75°
Reflection coefficient	0.7
Area of PD	10^−4^ m^2^
Responsivity of PD	0.5 A/W
Tx elevation	−90°
Tx azimuth	0°
Rx elevation	90°
Rx azimuth	0°

**Table 3:** The optimized values for all cases of Txs.

Number of Txs	Uniformity	Distance between Txs (m)	HPA (°)
4	0.65	2.4	60
9	0.55	1.3	60
16	0.65	1.34	40

## Data Availability

Not applicable.

## Nomenclature

ADOA	Angle difference of arrival
ANN	Artificial neural network
AOA	Angle of arrival
CDF	Cumulative distribution function
FOV	Field of view
GPS	Global positioning system
HPA	Half power angle
IoT	Internet of things
IP	Indoor positioning
LEDs	Light-emitting diodes
LLS	Linear least square
LOS	Line of sight
NLLS	Nonlinear least square
NLOS	Non-line of sight
OOK	On-off keying
PD	Photodiode
PDF	Probability distribution function
PR	Polynomial regression
RF	Radio frequency
RFID	Radio frequency identification
RSS	Received signal strength
Rx	Receiver
SNR	Signal-to-noise ratio
TDOA	Time difference of arrival
TOA	Time of arrival
Tx	Transmitter
UWB	Ultra-wide band
VLC	Visible light communication
VLP	Visible light positioning
